# Apis—Tonsilitis, Lach—Sore Throat

**Published:** 1881-05

**Authors:** William A. Hawley

**Affiliations:** Syracuse, N. Y.


### CLINICAL CASES.

Apis—Tonsilitis; Lach.—Sore Throat.

BY WILLIAM A. HAWLEY, M.D., SYRACUSE, N. Y.

APIS MELLIFICA.—Thursday, January 6th, 1881, late in the evening I was called to see Mr. P., aged about thirty. I found him suffering from tonsilitis, the right tonsil being so swollen and sore as to make deglutition of solids impossible and fluids almost so. The whole fauces looked very red, and the uvula was much swollen. He described the pains as stitching, especially on swallowing. I gave him Hepar Sulph. 30th, once in two hours. Calling the next morning I found he had had a restless night and was feeling no better. Examination of the throat by daylight showed uvula looking like a sack of water and he described the pains as "burning, stinging." I gave him Apis Mel. 70 M. (Fincke) a few pellets dissolved in half a tumbler of water, two teaspoonfuls once in four hours. The next morning, January 8th, I found he had rested well all night, and had eaten breakfast without difficulty. He got Sac. Lac. and resumed his business on Monday.

Jan. 13th, 1881. A gentleman called on me to prescribe for his wife who is very fleshy and three or four years past the climacteric. She had a swelling of the left labium vaginæ which he described as hard, hot and of a bluish red color. The side and the color determined the prescription and she got Lachesis 30th, in water, once in two hours.

On the 15th he reported not much change except that it did not seem quite as hard, and she got Lach. CC, in water, once in two hours.

The 17th he reported again that there was less soreness but more swelling and the heat and color about the same. Gave Lachesis C.M. once in four hours. Next day I saw the patient for the first time, and on examination found the swelling was not, as I had supposed, phlegmonous; but, while it was enormous, it was puffy and very sore when sitting, with a burning pain. This decided a change to Apis Mel. 70 C.M., in water; a dose once in four hours, which, in two days, ended the case.

LACHESIS.—One day last week a lady, unaccustomed to Homœopathic treatment, said to me during a casual conversation, "Doctor, I wish you would give me something for my throat. I have had a very troublesome throat for several weeks. There is a sore spot; I can cover it with my finger, so (putting the end of her finger on the left side of her neck just below the angle of the jaw). It feels as if there was a lump in it that I want all the time to swallow; and when I swallow, it prickles like needles in it." I gave her a few pellets of Lachesis CC., with directions to dissolve in a gill of water and take two teaspoonfuls and repeat after four hours, if not better. The next day I learned from her daughter that her throat was quite well. The fourth day after, I saw her and inquiring after her health, she replied, "my throat is quite well, but, doctor, was there any thing in that medicine that would make my gums sore?" I said, "Yes, if you have been mercurialized." She admitted that she had been under the free use of "blue mass."

How are the Milwaukee tests?

